# A correction for sample overlap in genome-wide association studies in a polygenic pleiotropy-informed framework

**DOI:** 10.1186/s12864-018-4859-7

**Published:** 2018-06-25

**Authors:** Marissa LeBlanc, Verena Zuber, Wesley K. Thompson, Ole A. Andreassen, Schizophrenia and Bipolar Disorder Working Groups of the Psychiatric Genomics Consortium, Arnoldo Frigessi, Bettina Kulle Andreassen

**Affiliations:** 1 Oslo Centre for Biostatistics and Epidemiology, Oslo University Hospital, Oslo universitetssykehus HF, Sogn Arena, PB 4950 Nydalen, Oslo, 0424 Norway; 2 MRC Biostatistics Unit, University of Cambridge, MRC Biostatistics Unit, Cambridge Institute of Public Health, Robinson Way, Cambridge, CB2 0SR United Kingdom; 3 Department of Psychiatry, University of California, San Diego, 9500 Gilman Drive, MC 0603, La Jolla, CA, 92093-0603 USA; 4 NORMENT-KG Jebsen Centre for Psychosis Research, Institute of Clinical Medicine, University of Oslo, P.O. Box 1039 Blindern, Oslo, N-0315 Norway; 5 Division of Mental Health and Addiction, Oslo University Hospital HF, Ullevaal Hospital, building 49,P.O. Box 4956 Nydalen, Oslo, N-0424 Norway; 6 Oslo Centre for Biostatistics and Epidemiology, University of Oslo and Oslo University Hospital, Oslo universitetssykehus HF, Sogn Arena, PB 4950 Nydalen, Oslo, 0424 Norway; 7 Department of Research, Cancer Registry of Norway, P.O. box 5313 Majorstuen, Oslo, N-0304 Norway

**Keywords:** Data integration, Meta-analysis with shared subjects, Covariate-modulated false discovery rate, Cross-phenotype association

## Abstract

**Background:**

There is considerable evidence that many complex traits have a partially shared genetic basis, termed pleiotropy. It is therefore useful to consider integrating genome-wide association study (GWAS) data across several traits, usually at the summary statistic level. A major practical challenge arises when these GWAS have overlapping subjects. This is particularly an issue when estimating pleiotropy using methods that condition the significance of one trait on the signficance of a second, such as the covariate-modulated false discovery rate (cmfdr).

**Results:**

We propose a method for correcting for sample overlap at the summary statistic level. We quantify the expected amount of spurious correlation between the summary statistics from two GWAS due to sample overlap, and use this estimated correlation in a simple linear correction that adjusts the joint distribution of test statistics from the two GWAS. The correction is appropriate for GWAS with case-control or quantitative outcomes. Our simulations and data example show that without correcting for sample overlap, the cmfdr is not properly controlled, leading to an excessive number of false discoveries and an excessive false discovery proportion. Our correction for sample overlap is effective in that it restores proper control of the false discovery rate, at very little loss in power.

**Conclusions:**

With our proposed correction, it is possible to integrate GWAS summary statistics with overlapping samples in a statistical framework that is dependent on the joint distribution of the two GWAS.

**Electronic supplementary material:**

The online version of this article (10.1186/s12864-018-4859-7) contains supplementary material, which is available to authorized users.

## Background

The past decade of genomic research has been shaped by the advent of low-cost, high throughput technology, enabling the examination of a large number of genetic variants, i.e. single nucleotide polymorphisms (SNPs), via the genome-wide association study (GWAS). The success of the GWAS approach has been limited however because SNPs identified by GWAS only capture a small fraction of the total heritability for any given complex trait. There is ongoing debate on how to detect this so-called ‘missing heritability’ [1, 2], including ideas based on integrating GWAS data across two or more traits which may share a polygenic signal (e.g. [3]). A shared polygenic signal may exist for traits with strong diagnostic overlap and this has motivated the formation of cross-trait GWAS consortia such as the Psychiatric Genetics Consortium including five psychiatric diseases, and the International Cancer Genome Consortium that aims at finding oncogenes that might drive cancer growth in different sites. Seemingly unrelated phenotypes may also have a shared polygenic signal if they partially share a common genetic basis, termed pleiotropy [4]. Pleiotropic effects have been statistically detected in cross-trait analysis of GWAS, including schizophrenia and blood lipids [3], prostate cancer and blood lipids [5], and psychiatric disorders [6].

A major statistical challenge encountered when integrating GWAS data across traits is the widespread re-use of subjects between GWA studies, leading to non-independent data sets. Power has been maximized by increasing sample sizes, often in the hundreds of thousands, via large meta-analysis conducted by worldwide consortia for complex traits such as coronary artery disease (CAD) [7], height [8] and blood pressure [9]. Second, phenotype definitions have become more specific and have moved towards endophenotypes (e.g. blood lipids [10]), which are often measured on the same set of individuals. This, together with the epidemiological overlap of many common diseases, has led to the re-use of subjects from one GWAS to another. For example, control samples have been re-used for several different case definitions, often by design. The Wellcome Trust Case Control Consortium (WTCCC) [11] is one such consortium adopting this strategy. As another example, cases for one trait have been included in quantitative trait studies (*e*.*g*. CAD [7] and blood lipids [10] and height [8]).

Addressing subject overlap is complicated by that fact that GWAS data is most often made available in form of summary statistics, i.e data over *n* samples is condensed into one summary statistic per SNP. GWAS summary statistics from studies with overlapping subjects cannot be made independent by removing these subjects. Aside from the issue of sample overlap, working on the summary statistics level has many advantages. When a sufficient statistic is used this summary statistic contains all the information necessary for further inference. Also, it is computationally efficient to work with summary statistics simply because of the much smaller size compared to the genotype data. This is especially relevant for the integration of several genomic data sets. Importantly, in contrast to genotype data, summary statistics cannot be used to uniquely identify individuals. This allows easier distribution and storage. As a consequence there are several consortia, such as the DIAGRAM Consortium for type 2 diabetes and the Global Blood Lipids Consortium, that have summary statistics covering the whole genome for free download on their homepage.

Lin and Sullivan [12] were the first to address the methodological challenge of integrating GWAS with overlapping subjects. Their contribution focused on integrating case-control GWAS using a meta-analysis framework. They do not provide a framework for integrating GWAS coming from different types of outcome variables (e.g. a case-control study and a quantitative trait study), nor do they provide a solution that applies in general to different statistical methodology. Han et al. [13] extend the Lin and Sullivan approach for cases and controls to random effects meta-analysis setting using a decoupling approach.

Two other approaches for meta-analysis of multiple traits while accounting for sample overlap are presented by [14, 15]. While these two approaches account for sample overlap in performing the meta-analysis, [16] introduce a test statistic based on a similar derivation as Lin and Sullivan that allows to test for overlapping samples or relatives when performing quality control of summary level data.

There is growing interest in statistical methods that utilize the joint bivariate distribution of GWAS summary statistics for two traits because, in the presence of a shared polygenic signal, these methods may provide more power than traditional GWAS methodology. One such method is the covariate-modulated local false discovery rate (cmfdr) proposed by Ferkingstad et al. [17] and recently revisited and extended [18] where the fdr for the first study depends on a covariate, for example the GWAS summary statistics for a second pleiotropic trait.

Similarly, the tail-area based conditional false discovery rate [3] needs the joint distribution of two sets of GWAS summary statistics to identify SNPs with cross-phenotype associations. These methods may be seriously impacted by the spurious correlation due to overlap, but cannot be corrected on a SNP-by-SNP basis. Liley and Wallace [19] extend the conditional false discovery rate [3] to studies with overlapping controls. Their extension is specific to case-control studies and does not apply to the cmfdr or any other bivariate method.

The aims of this paper are threefold. First, we want to show the impact of overlap in samples on integrated analyses of genetic studies. We show that it can induce spurious correlation between the studies and thus seriously confound conclusions. Second, we expand on the work of Lin and Sullivan [12] and quantify the spurious cross-trait correlation due to overlap for both case-control studies and studies with quantitative traits. And third, we propose a correction based on a decorrelation transformation that adjusts the joint distribution of two GWAS and allows for the use of the corrected summary statistics in downstream analysis such as cmfdr. We demonstrate the impact of overlap in samples and the success of our proposed correction on synthetic and GWAS data from the Psychiatric Genetics Consortium (PGC).

## Results

### The impact of overlap in samples on the joint analysis of two genomic data sets

The overlap of samples between two GWAS induces spurious correlation in a bivariate analysis of the two data sets. We illustrate this spurious correlation in a simulation example. The simulation is based on two studies, 1 and 2, with *d*=100,000 SNPs of a minor allele frequency (MAF) drawn at random from the allele frequency distribution in the 1000 Genomes Project [20]. Genotypes are generated under the null model of no genetic association and accordingly are drawn from a binomial distribution with 2 trials and probability of success equal to the MAF. Each study has a continuous outcome that only depends on the error term (normal with mean 0 and standard deviation of 1). Study 1 and study 2 have *n*_*C*_=5,000 shared subjects and *n*_*A*_=*n*_*B*_=7,500 unique subjects respectively. Thus the total sample size per study is *n*_1_=*n*_2_=12,500. We then conduct a standard GWAS analysis (univariate linear regression, one SNP at a time) separately in study 1 and study 2.

**Fig. 1:**
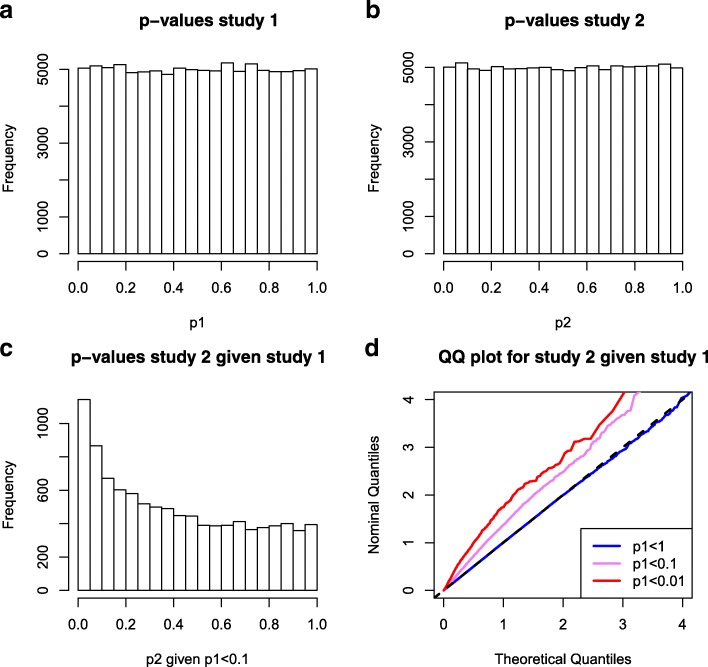
Simulated GWAS pairs with overlapping samples. Data was simulated for two quantitative trait GWAS with no genetic effects but overlap in samples (each with *n*=12,500 including 5000 overlapping samples). *d*=100,000 SNPs were simulated under the null model (phenotype is simulated independent from genotype). Panel **a**: the *p*-value distribution for trait 1; Panel **b**: the *p*-value distribution for trait 2; Panel **c**: The *p*-value distribution for trait 2 given that the *p*-value in study 1 was less than 0.1; Panel **d**: quantile-quantile plot for the *p*-values in study 2, stratified by the *p*-value in study 1

Figure 1a and b show that *p*-values for study 1 and for study 2 respectively follow a uniform distribution as expected. Assume we are interested in selecting the SNPs in study 2 on the basis of their significance in study 1. Figure 1c shows the *p*-values of study 2 for which the *p*-values in study 1 are smaller than 0.1. Finally, Fig. 1d displays a stratified Q-Q plot that plots the observed quantiles of the *p*-values of study 2 against the quantiles assumed under the null distribution. The strata are defined with respect to the *p*-values in study 1. These stratified Q-Q plots offer an intuitive way of visualizing dependencies between *p*-values of two different genetic studies. Despite being generated without any genetic effects, we observe that the conditional distributions of *p*-values from study 2 given *p*-values in study 1 show strong enrichment for small *p*-values with respect to the second conditional phenotype. If we were unaware that these simulations were conducted under the null hypothesis, this leftward deflection of the stratified Q-Q plot could be falsely interpreted as shared polygenic pleiotropic signal. Clearly, in case of overlapping samples, pleiotropic effects would be confounded with the spurious effects due to sample overlap.

### Estimating the correlation of two test statistics due to overlap in samples

Details of this estimation are given in the “Methods” section. Consider two studies, *k*=1,2, both with continuous outcomes, *y*_*ki*_, *i*=1,…,*n*_*k*_. Assume some samples are shared, so that we can split the set of samples {1,…,*n*_*k*_} into two sets *S*_*C*_={1,…,*n*_*c*_} and *S*_*A*_={*n*_*c*_+1,…,*n*_1_} for study 1 and similarily for study 2 with *S*_*B*_={*n*_*c*_+1,…,*n*_2_}. *S*_*C*_ are the shared samples and *S*_*A*_ and *S*_*B*_ are the samples unique to study 1 and study 2 respectively. The full set for study 1 is *S*_1_=*S*_*C*_∪*S*_*A*_ and for study 2 is *S*_2_=*S*_*C*_∪*S*_*B*_. Denote with *X*_*kig*_ the random genotypes for SNP *g* in sample *i* in study *k*, *g*=1,2,..,*d*, where *d* is typically some large number (≈10^6^). Simlarly, denote with *X*_*kjg*_ the random genotypes in sample *j*.Then, cor(*X*_1*ig*_,*X*_2*jg*_)=1 if *i*∈*S*_*C*_ for all SNPs *g* and we assume cor(*X*_1*ig*_,*X*_2*jg*_)=0 if *i*∈*S*_*A*_ and *j*∈*S*_*B*_ for all *g*.

Consider two regression models, one for each study for one SNP *g* at a time, *Y*_1*i*_=*α*_1*g*_+*β*_1*g*_*X*_1*ig*_+*ε*_1*ig*_ and *Y*_2*j*_=*α*_2*g*_+*β*_2*g*_*X*_2*jg*_+*ε*_2*jg*_ where *i*=1,..,*n*_1_, *j*=1,..,*n*_2_, and we assume all errors *ε* to be independent from each other and with zero mean. Under the null model (*β*_*kg*_=0) ∀*k*,*g*, if *S*_*C*_ was an empty set (i.e. no shared subjects), then $\text {cor}\left (\hat {\beta }_{1g},\hat {\beta }_{2g}\right) = 0$. But because of the shared samples *S*_*C*_, $\rho = \text {cor}\left (\hat {\beta }_{1g},\hat {\beta }_{2g}\right) \neq 0$, the overlap between samples introduces a correlation of the regression parameters which is only due to the overlap. Note, when analyzing study 1 and study 2 separately the analysis is unbiased; the bias due to overlap is only introduced in a joint analysis where *ρ*≠0 is neglected, as illustrated in Fig. 1.

Building on the work of Lin and Sullivan [12], we estimate the correlation *ρ* due to overlap in samples under the null model (*β*_*kg*_=0) ∀*k*,*g*, using the correlation between the maximum likelihood (ML) estimates for the regression coefficients for SNP *g* denoted by $\hat {\beta }_{kg}$. The ML estimates are asymptotically Gaussian distributed with mean equal to the true coefficients *β*_*kg*_ and variance equal to the inverse Fisher information.

We are also interested in combined analysis of GWAS summary statistics from other study designs, including those analyzed in a case-control study. Therefore, in the following we estimate *ρ* for three possible scenarios with (*Y*_1_ and *Y*_2_ both quantitative; *Y*_1_ quantitative and *Y*_2_ binary; *Y*_1_ and *Y*_2_ both binary, where $Y_{k}=\{Y_{k1},Y_{k2},\ldots,Y_{k{n_{k}}}\}\phantom {\dot {i}\!}$ for *k*=1,2). The ML-based derivations (see “Methods” section) result in the following estimated correlation due to sample overlap for each of the three possible study design pairings:

1. Quantitative phenotype in both study 1 and study 2. For each SNP *g*,
  1 $$  \text{cor}(\hat{\beta}_{1g}, \hat{\beta}_{2g}) \approx \frac{n_{c}}{\sqrt{n_{1} \cdot n_{2}}} \text{cor}(Y_{1}, Y_{2})  $$
  where *n*_*c*_ is the number of overlapping samples in study 1 and 2, *n*_1_ is the sample size of study 1, and *n*_2_ the sample size of study 2, respectively. Note that under the null hypothesis of no SNP effect, this correlation does not depend on the MAF and is the same for every SNP. In this case the *g* subscript can be dropped and $\text {cor}\left (\hat {\beta }_{1g},\hat {\beta }_{2g}\right)$ can instead be written as $\text {cor}\left (\hat {\beta }_{1},\hat {\beta }_{2}\right)$, and this simplified notation is used from this point on.
2. Binary phenotype in study 1 and binary phenotype in study 2
  2 $$ {\selectfont{\begin{aligned} {}\text{cor}\left(\hat{\beta}_{1}, \hat{\beta}_{2}\right)\! \!\approx\! \frac{1}{\sqrt{n_{1}}\sqrt{n_{2}}} \!\times\! \left(n_{c0}\sqrt{\exp \{\alpha_{1} + \alpha_{2}\}} + \frac{n_{c1}}{\sqrt{\exp \{\alpha_{1} + \alpha_{2}\}}} \right) \end{aligned}}}  $$
  where exp{*α*_1_+*α*_2_}≈*n*_11_*n*_21_/*n*_10_*n*_20_ [12] and where we denote the number of cases in study 1 and 2 as *n*_11_ and *n*_21_ respectively, similarly *n*_10_ and *n*_20_ for the number of controls in study 1 and 2 respectively, and denote the overlap in controls by *n*_*c*0_ and in cases by *n*_*c*1_.
3. Quantitative phenotype in study 1 and binary phenotype in study 2
  3 $$  cor\left(\hat{\beta}_{1}, \hat{\beta}_{2}\right) \approx \frac{n_{c}}{\sqrt{n_{1} \cdot n_{2}}} \text{cor}_{pb}(Y_{1}, Y_{2})  $$
  where cor_*pb*_(*Y*_1_,*Y*_2_) equals the point-biserial correlation coefficient.

Note that the estimates $cor\left (\hat {\beta }_{1},\hat {\beta }_{2}\right)$ in Eqs. 1 to 3 only estimate the spurious correlation due to sample overlap. This estimate differs from the total correlation between the observed test statistics which captures both the true correlation based on genetic architecture and the spurious correlation induced by sample overlap.

### Decorrelation using the correlation due to overlap

In this paper we propose a decorrelation step to adjust the joint distribution of the summary statistics from two GWAS having overlapping subjects. Construct a matrix **z** consisting of two rows and *d* columns equal to the number of SNPs common to both studies, including the vector of summary statistics (*z*-scores) for the first study, *z*_1_, in the first row and the vector of *z*-scores for the second study, *z*_2_, in the second row. The decorrelation transform is defined as

4 $$  \mathbf{z_{\text{de-corr}}} = \mathbf{C}^{-1/2} \mathbf{z}  $$

where **C** is the 2×2 matrix with ones on its diagonal the calculated correlation due to overlap on its off-diagonal.

**Fig. 2:**
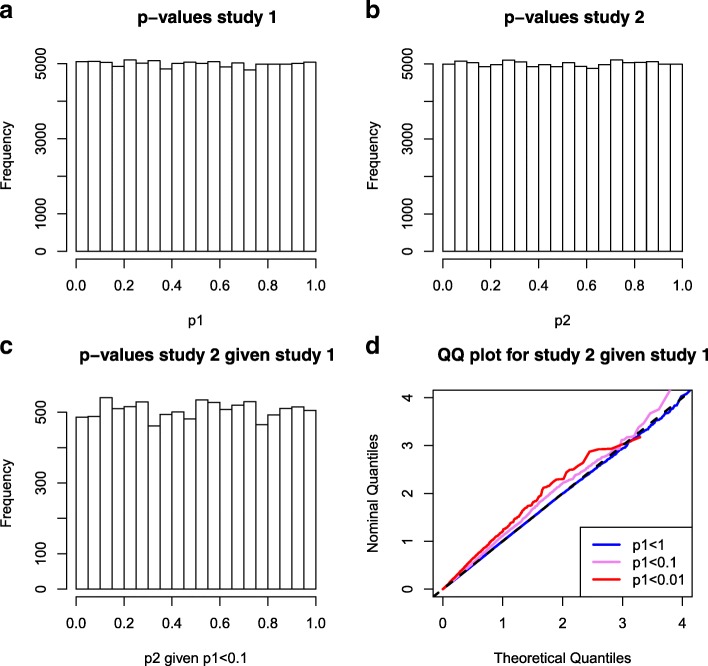
Simulated GWAS pairs with overlapping samples, after correction for sample overlap using the decor relation transform. Data before correction is presented in Fig. 1. Data was simulated for two quantitative trait GWAS with no genetic effects but overlap in samples ((each with *n*=12,500 including 5,000 overlapping samples). *d*=100,000 SNPs were simulated under the null model (phenotype is simulated independent from genotype). The decor relation transformation proposed here was applied to the simulated summary statistics. Panel **a**: the *p*-value distribution for trait 1; Panel **b**: the *p*-value distribution for trait 2; Panel **c**: The *p*-value distribution for trait 2 given that the *p*-value in study 1 was less than 0.1; Panel **d**: quantile-quantile plot for the *p*-values in study 2, stratified by the *p*-value in study 1

In Fig. 2 we use the simulated data introduced in Fig. 1 and show how the proposed decorrelation step corrects for the correlation due to overlap and removes the spurious enrichment. The *p*-values for study 2 conditional on study 1 are equally distributed (Fig. 2c) and the inflation of the enrichment is removed (Fig. 2d).

### Performance of proposed decorrelation step in a covariate-modulated false discovery rate framework

We tested the performance of our proposed correction for sample overlap in a covariate-modulated fdr (cmfdr) [18] framework using a two-pronged approach. First, we quantified the impact of sample overlap on the actual false discovery proportion under different pleiotropic simulation scenarios and with different amounts of sample overlap. Second, we used individual-level (genotype-phenotype) data from the Psychiatric Genetics Consortium (PGC), which employed a shared control design for schizophrenia and bipolar disorder, to test our correction in a real data setting. Since we had access to the individual-level data, we were able to conduct a series of GWAS manipulating the extent of overlapping controls and compare the number of cmfdr-based “discoveries” to equally-powered non-overlapping control sets.

#### Simulated data

We simulated bivariate GWAS data under six different simulation scenarios: first under the null model, where genotype is independent from phenotype and then under five different pleiotropic scenarios:

1. Null model, no effect
2. Positive pleiotropy A
3. Positive pleiotropy B
4. Positive pleiotropy C
5. Positive pleiotropy plus univariate effects
6. Positive and antagonistic pleiotropy,

where positive pleiotropy A, B and C differ in the extent of polygenic structure.

We then used this simulated data to conduct synthetic GWAS for paired studies with first no sample overlap and then again with sample overlap. For each study pair, we calculated the cmfdr for the first GWAS using the summary statistics from the second GWAS as a covariate. We did this both with and without our proposed correction for sample overlap and compared the false discovery proportion (FDP), i.e. the number of false discoveries divided by the total number of discoveries, before and after correction and to the non-overlapping GWAS.

##### Simulation results

The main purpose of the simulation was to test the performance of our correction for sample overlap in a cmfdr framework with known null and non-null SNPS under different pleiotropic and polygenic scenarios and with different amounts of sample overlap.

Table 1 reports the mean false discovery proportion (FDP), mean number of falsely rejected null hypotheses (i.e. false positives (FP)) and mean number of correctly rejected non-null hypotheses, (i.e. true positives (TP)) under different simulation scenarios with *d*=100,000 SNPs over independent 100 simulations based on a cmfdr cutoff of 0.05 and using the summary statistics from study 2 as a covariate for study 1. This is reported for all six simulation scenarios. The null model simulation shows that, in the absence of any true genetic association and with non-overlapping samples, no SNPs reach the cmfdr cutoff of 0.05. In contrast, when samples overlap, a mean of 245 SNPs are below the cutoff, and thus are false positives. After applying our proposed correction to the GWAS with overlapping samples, all cmfdr values are again above the significance cutoff and no SNPs are deemed significant. For the simulation scenarios involving pleiotropic effects, 400 of the 100,000 SNPs were non-null except for positive pleiotropy B and C where 1200 and 2200 were non-null respectively. For all pleiotropic scenarios, the FDP for the analysis using the non-overlapping studies shows that the fdr level is conservatively held, while the FDP for the overlapping set, greatly exceeds the desired level of fdr control. After correction the overlapping studies using the proposed decorrelation step, the fdr control is comparable to the non-overlapping, independent studies.

**Table 1:** Mean false discovery proportion (FDP), mean number of falsely rejected null hypotheses out of 99,600, i.e. false positives (FP) and mean number of correctly rejected non-null hypotheses i.e. true positives (TP) over 100 simulation runs and a covariate-modulated false discovery rate (cmfdr) cut-off of 0.05

Model	Independent	Independent, eq. power	Overlapping	Overlapping, corrected
Null				
FDP	–	–	–	–
TP	–	–	–	–
FP	0	0	245.14 (237.9, 252.4)	0
Positive Pleiotropy A (400 non-null SNPs)				
FDP	0.0053 (0.0044, 0.0063)	0.0030 (0.0024, 0.0037)	0.39 (0.39, 0.40)	0.0056 (0.0048, 0.0065)
TP	260.9 (259.6, 262.1)	283 (282.0, 284.1)	330.4 (329.5, 331.4)	243.4 (242.1, 244.7)
FP	1.4 (1.2, 1.7)	0.9 (0.7, 1.1)	215.7 (210.1, 221.4)	1.4 (1.2, 1.6)
Positive Pleiotropy + Univariate (400 non-null SNPs)				
FDP	0.008 (0.007, 0.009)	0.005 (0.004, 0.006)	0.48 (0.48, 0.49)	0.01 (0.008, 0.01)
TP	233.4 (232.1, 234.7)	270.4 (269.4, 271.5)	306.1 (304.7, 307.4)	209.8 (208.4, 211.2)
FP	2.0 (1.7, 2.2)	1.3 (1.1, 1.5)	289.2 (282.4, 296.1)	2.08 (1.8, 2.4)
Positive + Antagonistic Pleiotropy (400 non-null SNPs)				
FDP	0.005 (0.005, 0.006)	0.004 (0.003, 0.005)	0.46 (0.45, 0.47)	0.008 (0.007, 0.010)
TP	261.5 (260.4, 262.6)	290.8 (289.6, 291.9)	280.9 (280.0, 282.2)	228.7 (227.3, 230.1)
FP	1.4 (1.2, 1.6)	1.2 (1.0, 1.4)	240.1 (233.8, 246.4)	2.0 (1.7, 2.2)
Positive Pleiotropy B (1200 non-null SNPs)				
FDP	0.018 (0.008, 0.020)	0.013 (0.012, 0.014)	0.32 (0.31, 0.33)	0.029 (0.027. 0.031)
TP	295.65 (293.01, 298.29)	425.38 (422.42, 428.34)	618.30 (615.22, 621.38)	310.94 (308.22, 313.66)
FP	5.51 (5.05, 5.97)	5.60 (5.11, 6.09)	294.64 (288.18, 301.10)	9.36 (8.70, 10.02)
Positive Pleiotropy C (2200 non-null SNPs)				
FDP	0.019 (0.017, 0.021)	0.018 (0.016, 0.020)	0.36 (0.35, 0.36)	0.034 (0.032, 0.037)
TP	159.71 (157.63, 161.79)	243.98 (241.91, 246.04)	575.33 (570.58, 580.08)	184.10 (181.68, 186.52)
FP	3.16 (2.80, 3.52)	4.49 (4.05, 4.92)	324.94 (317.67, 332.20)	6.59 (6.08, 7.10)

Results are presented for six different simulation scenarios: the *null model*, where both traits are independent from genotype (all SNPs are null); *positive pleiotropy A* with 400 SNPs that are non-null for both traits; *positive pleiotropy plus univariate effects for trait 1*, where 200 SNPs were non-null for traits 1 and 2 and 200 SNPs were non-null for trait 1 only; *positive plus antagonistic pleiotropy*, where 400 SNPs were non-null for both traits 1 and 2, and half of these non-null SNPs have an effect in opposing directions for trait 1 and 2; *positive pleiotropy B* with 1200 SNPs that are non-null for both traits, 200 with large effects and 1000 with small effects; *positive pleiotropy C* with 2200 SNPs that are non-null for both traits, 200 with large effects and 2000 with small effects. In all six scenarios *d*=100,000 SNPs were simulated, the correlation due to overlap is 0.4 and the test statistics for study 2 were used as a covariate for study 1 for the covariate-modulated fdr. For each simulation scenario, we divided the simulated subjects into the following GWAS pairs: ***Independent***, independent GWASs with no overlap (each with *n*=10,000), ***Independent eq. power***, independent equally-powered GWASs (each with *n*=12,500 like the GWASs with overlapping subjects), ***Overlapping***, uncorrected overlapping GWAS with (each with *n*=12,500 including,5000 overlapping, subjects) and ***Overlapping, corrected***, the GWAS with 5,000 overlapping subjects after correction for sample overlap. Data is presented as mean (95% confidence interval)

**Fig. 3:**
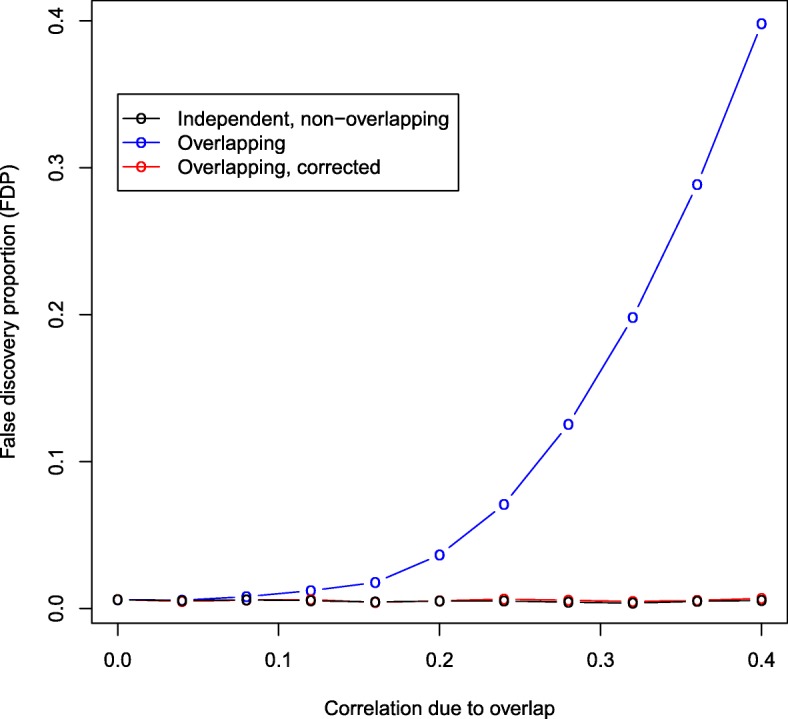
Mean false discovery proportion (FDP) versus the correlation due to sample overlap over 100 simulation runs and a covariate-modulated false discovery rate (cmfdr) cut-off of 0.05. Here *d*=100,000 SNPs were simulated, of which 400 were non-null in both study 1 and study 2, i.e., have positive pleiotropic effects. The test statistics for study 2 were used as a covariate for study 1

We performed an extended simulation using the “positive pleiotropy A” scenario, where we varied the amount of sample overlap. Table 2 and Fig. 3 give the FDP, TP and FP and clearly show that the impact of sample overlap is non-linear. The FDP increases at an increasing rate as the number of overlapping samples increases. After applying our correction for sample overlap to the overlapping studies, the fdr control is comparable to the non-overlapping, independent studies for all levels of sample overlap. The correction results in a small loss in power (TP), and this loss in power is more severe as the overlap increases.

**Table 2:** Mean false discovery proportion (FDP), mean number of falsely rejected null hypotheses out of 99,600, i.e. false positives (FP) and mean number of correctly rejected non-null hypotheses out of 400 s, i.e. true positives (TP) over 100 simulation runs and a covariate-modulated false discovery rate (cmfdr) cut-off of 0.05

#	*ρ*		Independent	Overlapping	Overlapping, corrected
0	0				
		FDP	5.96E-03 (4.99E-03, 6.92E-03)	5.92E-03 (4.97E-03, 6.88E-03)	6.03E-03 (5.07E-03,7.00E-03)
		TP	268.55 (267.90, 269.90)	268.52 (267.18, 269.86)	268.59 (267.18, 269.86)
		FP	1.62 (1.36, 1.88)	1.61 (1.35, 1.87)	1.64 (1.37, 1.91)
500	0.04				
		FDP	5.32E-03 (4.41E-03,6.22E-03)	5.58E-03 (4.58E-03,6.59E-03)	4.77E-03 (3.81E-03,5.73E-03)
		TP	262.75 (261.58,263.92)	266.3 (264.99, 267.61)	260.47 (259.05, 261.61)
		FP	1.41 (1.17, 1.65)	1.5 (1.23, 1.77,)	1.25 (1.00, 1.50)
1000	0.08				
		FDP	5.83E-03 (4.87E-03, 6.78E-03)	8.02E-03 (6.81E-03, 9.23E-03)	5.69E-03 (4.76E-03,6.63E-03)
		TP	263.43 (262.08, 264.78)	271.85 (270.59, 273.11)	258.92 (257.57, 260.27)
		FP	1.55 (1.29, 1.81)	2.21 (1.87, 2.55)	1.49 (1.24, 1.74)
1500	0.12				
		FDP	5.25E-03 (4.44E-03, 6.06E-03)	1.21E-02 (1.08E-03, 1.34E-02)	6.00E-03 (5.08E-03, 6.92E-03)
		TP	263.67 (262.43, 264.91)	277.11 (275.82, 278.40)	257.79 (256.51, 259.07)
		FP	1.4 (1.18, 1.62)	3.4 (3.02, 3.78)	1.56 (1.32, 1.80)
2000	0.16				
		FDP	4.42E-03 (3.61E-03, 5.22E-03)	1.77E-02 (1.61E-02, 1.92E-02)	4.06E-03 (3.26E-03, 4.86E-03)
		TP	255.16 (253.98, 256.34)	274.18 (273.10, 275.26)	248.52 (247.27, 249.77)
		FP	1.14 (0.93, 1.35)	4.96 (4.51, 5.41)	1.02 (0.82, 1.22)
2500	0.20				
		FDP	5.03E-03 (4.16E-03,5.90E-03)	3.64E-02 (3.38E-02, 3.91E-02)	5.20E-03 (4.28E-03, 6.12E-03)
		TP	258.84 (257.51, 260.17)	288.47 (287.29, 289.65)	249.59 (248.22, 250.96)
		FP	1.31 (1.08, 1.54)	10.98 (10.15, 11.81)	1.31 (1.08, 1.54)
3000	0.24				
		FDP	5.08E-03 (4.18E-03, 5.97E-03)	7.08E-02 (6.74E-02,7.42E-02)	6.32E-03 (5.41E-03, 7.22E.03)
		TP	261.65 (260.32, 262.98)	300.52 (299.39, 301.65)	250.14 (248.75, 251.53)
		FP	1.34 (1.10, 1.58)	23.03 (21.83, 24.23)	1.6 (1.37, 1.83)
3500	0.28				
		FDP	4.24E-03 (3.52E-03, 4.96E-03)	1.25E-01 (1.21E-01, 1.30E-01)	5.57E-03 (4.74E-03, 6.40E-03)
		TP	268.5 (267.37, 269.63)	315.07 (314.00, 316.14)	256.42 (255.08, 257.76)
		FP	1.15 (0.95, 1.35)	45.42 (43.46, 47.38)	1.44 (1.23, 1.65)
4000	0.32				
		FDP	3.62E-03 (2.84E-03, 4.41E-03)	1.98E-01 (1.93E-01, 2.03E-01)	4.74E-03 (3.91E-03, 5.56E-03)
		TP	262.39 (261.27, 263.51)	316.5 (315.46, 317.54)	249.16 (247.94, 250.38)
		FP	0.96 (0.75, 1.17)	78.65 (76.05, 81.25)	1.19 (0.98, 1.40)
4500	0.36				
		FDP	4.81E-03 (3.99E-03, 5.63E-03)	2.89E-01 (2.83E-01, 2.94E-01)	5.49E-03 (4.54E-03, 6.44E-03)
		TP	259.29 (258.16, 260.42)	319.99 (319.04, 320.94)	245.16 (243.92, 246.40)
		FP	1.26 (1.04, 1.48)	130.41 (127.08, 133.74)	1.36 (1.12, 1.60)
5000	0.40				
		FDP	5.44E-03 (4.57E-03, 6.31E-03)	3.98E-01 (3.92E-01, 4.04E-01)	6.79E-03 (5.78E-03, 7.80E-03)
		TP	262.26 (261.02, 263.50)	334.25 (333.28, 335.22)	245.02 (243.66, 246.38)
		FP	1.44 (1.21, 1.67)	222.52 (216.73, 228.31)	1.68 (1.43, 1.93)

Here *d*=100,000 SNPs were simulated, of which 400 were non-null in both study 1 and study 2, i.e., the positive pleiotropy senario. The test statistics for study 2 were used as a covariate for study 1 for the covariate-modulated fdr. For each simulation, we divided the simulated subjects into the following GWAS pairs: ***Independent***, independent GWASs with no overlap (each with *n*=10,000), ***Overlapping***, uncorrected, overlapping GWAS with (each with including between 0 and 5000 overlapping subjects) and ***Overlapping, corrected***, the GWAS with overlapping subjects after correction for sample overlap. Data is presented as mean (95% confidence interval)

#, number overlapping. *ρ*, correlation due to overlap

In practice it may be difficult to calculate the exact overlap in samples or obtain an accurate estimate of *Cor*(*Y*_1_,*Y*_2_) for continuous traits. We therefore tested the robustness of our proposed correction to the correlation used in the decorrelation step (Eq. 4). Using the positive pleiotropy A scenario, where $cor\left (\hat {\beta }_{1},\hat {\beta }_{2}\right)=0.4$, we varied the correlation value used in Eq. 4 from 0.3 to 0.5. We find that our proposed correction is robust the the correlation value used in the decorrelation step with fdr level being conservatively held in all cases (Table 3).

**Table 3:** Robustness of the proposed correction

True correlation	Plug-in correlation	TP	FP	FDP
0.4	0.3	261.16 (260.27, 262.85)	2.42 (2.12, 2.71)	0.0091 (0.0080, 0.0102)
0.4	0.35	252.20 (250.92, 253.48)	1.56 (1.32, 1.80)	0.0061 (0.0052, 0.0070)
0.4	0.375	247.78 (246.79, 249.06)	1.48 (1.22, 1.73)	0.0059 (0.0049, 0.0069)
0.4	0.4	243.59 (242.31, 244.879)	1.40 (1.17, 1.63)	0.0057 (0.0048, 0.0066)
0.4	0.425	238.72 (237.42, 240.02)	1.60 (1.38, 1.82)	0.0066 (0.0057, 0.0075)
0.4	0.45	235.11 (233.88, 236.34)	1.96 (1.72, 2.20)	0.0082 (0.0072, 0.0092)
0.4	0.5	234.81 (233.57, 236.04)	1.96 (1.72, 2.20)	0.0082 (0.0072, 0.0092)

For the “positive pleiotropy A” scenario the correlation due to overlap is 0.4. Here we varied the correlation value in the de-correlation step from 0.3 to 0.5. TP, true positives; FP false positives, FDP, false discovery proportion

#### Psychiatric Genetics Consortium (PGC) data with shared controls

We used the PGC data [21, 22] to test the performance of our proposed correction for sample overlap in a real data setting, where we varied the amount of overlap in the control group between the schizophrenia and bipolar studies, corresponding to an expected correlation of *ρ* = 0,0.09,0.18,0.27,0.36,0.45. Using this series of GWAS summary statistics for bipolar disorder and schizophrenia, we calculated the cmfdr using the bipolar disorder summary statistics as the covariate for schizophrenia. The cmfdr calculations were done for both the raw data and also for the data after correction for sample overlap.

##### PGC results

Which SNPs are null and which SNPs are non-null is unknown, so it is not possible to count the true and false positives. Instead, we can count the total number of SNPs below a given cmfdr threshold (TP+FP), and use the non-overlapping set as a reference point. In this case, we used a threshold of 0.05 and called all SNPs with a cmfdr below this threshold a discovery. Importantly, the number of controls is held constant across the different amounts of sample overlap. This rules out any differences in (TP+FP) that may be expected to due differences in power. There were on average 255 discoveries for the analysis with no overlapping controls and significantly more discoveries were made when samples overlapped, as is evident by the non-overlapping confidence intervals for the no overlapping controls scenario versus all overlapping scenarios (Table 4). After correction for sample overlap, the number of discoveries returned to a more comparable level, usually falling just below the number of discoveries made in the non-overlapping analysis.

**Table 4:** Psychiatric Genetics Consortium data, with varying amounts of overlapping controls

#Overlapping	Correlation	#Discoveries, raw	#Discoveries, adjusted
0	0	255.3 (239.8,270.8)	256.5 (239.7,273.3)
2000	0.09	322.3 (310.1,334.5)	206.5 (190.1, 222.9)
4000	0.18	479 (437.4,520.6)	194.5 (172.8, 216.2)
6000	0.27	827.6 (762.1, 893.1)	186.4 (162.9, 209.9
8000	0.36	1442.7 (1325.2, 1560.2)	188.9 (156.8, 221.0)
10000	0.45	2985.7 (2785.6, 3185.8)	212.7 (181.3, 244.1)

The test statistics for bipolar disorder were used as a covariate for schizophrenia in the covariate-modulated fdr (cmfdr). SNPs having a cmfdr < 0.05 were called as discoveries. Data is presented as mean (95% confidence interval)

## Discussion

There is an increasing interest in combining GWAS data over multiple traits, often using data at the summary statistics level. Here we have proposed a practical and generally applicable approach for estimating the amount of correlation in the test statistics for two GWASs having overlapping subjects and having any type of outcome variable. Using simulation studies assuming various genetic architecture models, we have quantified the magnitude of the effect of sample overlap on the covariate-modulated fdr and have shown that sample overlap can greatly increase the false discovery proportion (FDP). Our proposed correction for sample overlap, which is an efficient prewhitening transformation, restores the FDP to a comparable level to simulated scenarios with no sample overlap. Using data for bipolar disorder and schizophrenia from the Psychiatric Genetics Consortium, we show that increasing numbers of shared controls result in an increased number of “discoveries”, but these so-called discoveries are most likely false positives and indicate a loss of proper control of the false discovery rate.

Statistical methods for integrating GWAS data at the summary statistic level are well established. Examples of such methods are Fisher’s method [23], inverse-variance meta-analysis [23], the conjunctional false discovery rate [3], the covariate-modulated fdr [18] and Mendelian randomization [24]. These methods universally assume independent samples. Violation of this assumption will result in increased Type 1 error and biased effect estimates [24]. Lin and Sullivan [12] were the first to recognize this importance of the sample overlap problem in the context of cross-trait analysis of GWAS data. Their work is focused on correcting for sample overlap for case-control studies in the context of fixed-effects meta-analysis test statistics. Under the null hypothesis of no genetic effects, they derived the correlation between the maximum likelihood estimates for the logistic regression coefficients for a given SNP in study 1 and study 2 when there are partially overlapping subjects in case-control studies. Here we use the same approach to derive the correlation for a case-control GWAS paired with a quantitative trait GWAS, or for 2 quantitative trait GWASs. The spurious correlation due to sample overlap is derived under the null and quantifies the correlation which is solely induced by sample overlap and independent of any genetic effect. Others have recognized that the number of overlapping samples is not always known and have proposed methods for estimating the correlation due to overlap using summary statistics alone [14, 25]. These methods could be used for quantitative trait GWASs where in practice the correlation of the two phenotypes (*Cor*(*Y*_1_,*Y*2)) may be difficult to estimate. Our simulations show that our proposed correction is robust with respect to the assumed correlation due to overlap. Further, the impact of *Cor*(*Y*_1_,*Y*2) on the correlation due to overlap increases as the extent of overlap increases. In these cases it may be feasible to request an estimate of *Cor*(*Y*_1_,*Y*2) from the relevant GWAS consortium. Regardless of which method is used to derive the correlation induced by sample overlap, here we propose a general framework to account for this spurious correlation in a simple and yet efficient preprocessing step. Spurious correlation between test statistics can be introduced not only by sample overlap, but also by including relatives in both studies. This results in an effective number of overlapping samples a concept introduced in [16]. Our approach can be easily extended to account for the effective number of overlapping samples in replacing *n*_*c*_ by the effective number of overlapping samples.

## Conclusions

Our goal was to provide a more general solution to the problem of cross-trait integration of GWAS that could be applied to statistical methods depending on the joint distribution of 2 GWASs. It is a practical approach in that it is easy to implement and results in transformed test statistics that can be used in different data integration methods. We show that in a cmfdr setting, our correction properly maintains fdr control.

Here we have contributed to the growing body of evidence showing that sample overlap needs to be taken into account when integrating data across different traits. We have shown that our flexible and adaptable adjustment for sample overlap works well as shown with both simulation and with real data in the context of the cmfdr.

## Methods

### Derivation of the estimates for correlation due to overlap

The correlation due to overlap in samples is derived from the correlation of the maximum likelihood (ML) estimates of the regression coefficients between two studies under the assumption of no genetic effect. We focus on one regression per SNP *g* and include the intercept and no other covariates. Focusing first on quantitative outcomes, consider two linear regressions, for one SNP *g* (we drop the index *g*), *Y*_*k*_=*α*_*k*_+*β*_*k*_*X*_*k*_+*ε*_*k*_. We assume all errors *ε*_*k*_ to be independent from each other and with zero mean.

Lin and Sullivan [12] show that for two case control studies the covariance between the ML estimates of the logistic regression coefficients from study 1 and 2 can be approximated as $Cov\left (\hat {\beta }_{1}, \hat {\beta }_{2}\right) \approx I_{1}^{-1}(\beta _{1}) Cov (U_{1}(\beta _{1}), U_{2}(\beta _{2})) I_{2}^{-1}(\beta _{2})$ where *U*_*k*_ and *I*_*k*_ are the score function and Fisher’s information with respect to *β*_*k*_. We use the above to further define the following correlation:

5 $$ {} Cor \left(\hat{\beta}_{1}, \hat{\beta}_{2}\right) \!\approx\! I_{1}^{-1/2}(\beta_{1}) Cov (U_{1}(\beta_{1}), U_{2}(\beta_{2})) I_{2}^{-1/2}(\beta_{2}).   $$

It is now straightforward to expand this result to include quantitative trait studies using the ML estimates from linear regression.

For linear regression the score function with respect to *β*_*k*_ is given by $U(\beta _{k}) = \frac {1}{\sigma _{k}^{2}} \sum _{i \in S_{k}} (y_{ki} -(\alpha _{k} + \beta _{k} x_{ki})) x_{ki}$ and the Fisher information is given by $I(\beta _{k}) = \frac {1}{\sigma _{k}^{2}} \sum _{i \in S_{k}} x_{ki} x_{ki}$. Similarly for logistic regression the score function with respect to *β*_*k*_ is given by $U(\beta _{k}) = \sum _{i \in S_{k}} \left (y_{ki} -\frac {\exp \{\alpha _{k} + \beta _{k} x_{ki}\}}{1+\exp \{\alpha _{k} + \beta _{k} x_{ki}\}}\right) x_{ki}$ and the Fisher information is given by $I(\beta _{k}) = \sum _{i \in S_{k}} \frac {\exp \{\alpha _{k} + \beta _{k} x_{ki}\}}{(1+\exp \{\alpha _{k} + \beta _{k} x_{ki}\})^{2}} x_{ki} x_{ki}$.

We make the following assumptions:

1. *Y*_*k*_ is independent of *X*_*k*_, that is we assume the null model where there is no genetic effect in the data and *β*_*k*_=0 for all SNPs, *k*=1,2.
2. The overlapping samples have the same genotype in each study *x*_1*i*_=*x*_2*i*_ for *i*∈*S*_*C*_ for all SNPs.
3. Construct a variable *H* defined as $H = E \left (X_{k}X_{k}^{T}\right)$. We can estimate *H* under the null hypothesis and the following three estimates of *H* are approximately equal $ n_{1}^{-1} \sum _{i \in S_{1}} x_{1i} x_{1i} \approx n_{2}^{-1} \sum _{i \in S_{2}} x_{2i} x_{2i} \approx n_{C}^{-1} \sum _{i \in S_{C}} x_{1i} x_{2i}$.

In case-control studies we assume *y*_1*i*_=*y*_2*i*_ for *i*∈*S*_*C*_ (in other words cases in study 1 are cases in study 2). Thus *Cor*(*Y*_1_,*Y*_2_)=1 for the overlapping samples in case-control studies. For quantitative phenotypes we assume that we are able to derive appropriate estimates for *Cor*(*Y*_1_,*Y*_2_) from epidemiology studies.

### Correction for overlapping samples in studies with quantitative traits

In Eq. (5) we use the score function and the Fisher information derived in the linear regression model and arrive at

6 $$\begin{array}{@{}rcl@{}} Cor(\hat{\beta}_{1},\hat{\beta}_{2}) &\approx& \left(\frac{1}{\sigma^{2}_{1}} \sum\limits_{i \in S_{1}} x_{1i} x_{1i}\right)^{-1/2}  \\ & & \times \frac{1}{\sigma^{2}_{1}}\frac{1}{\sigma^{2}_{2}} \frac{1}{n_{c}} \sum\limits_{i \in S_{C}} (y_{1i} - \alpha_{1}) (y_{2i} - \alpha_{2}) x_{1i} x_{2i}  \\ & & \times \left(\frac{1}{\sigma^{2}_{2}} \sum\limits_{i \in S_{2}} x_{2i} x_{2i}\right)^{-1/2}. \end{array} $$

Assumption 2 allows us to replace the sums over *x*_*ki*_ with *H* so $Cor\left (\hat {\beta }_{1},\hat {\beta }_{2}\right) \approx (n_{1} H)^{-1/2} \times \frac {1}{\sigma _{1}}\frac {1}{\sigma _{2}} H \sum \limits _{i \in S_{C}} (y_{1i} - \alpha _{1}) (y_{2i} - \alpha _{2}) \times (n_{2} H)^{-1/2}$, which simplifies to $Cor\left (\hat {\beta }_{1},\hat {\beta }_{2}\right) \approx \frac {1}{\sqrt {n_{1}}\sqrt {n_{2}}} \times \frac { \sum \limits _{i \in S_{C}}{(y_{1i} - \alpha _{1}) (y_{2i} - \alpha _{2})}}{\sigma _{1} \cdot \sigma _{2}}$. Multiplying by *n*_*c*_/*n*_*c*_ we get: $Cor\left (\hat {\beta }_{1},\hat {\beta }_{2}\right) \approx \frac {n_{c}}{\sqrt {n_{1}}\sqrt {n_{2}}} \times \frac { \frac {1}{n_{c}} \sum \limits _{i \in S_{C}} (y_{1i} - \alpha _{1}) (y_{2i} - \alpha _{2})}{\sigma _{1} \cdot \sigma _{2}}$. When individual level data is available, this can be computed directly. But when only summary statistics are available, the correlation can be approximated as

7 $$ Cor\left(\hat{\beta}_{1},\hat{\beta}_{2}\right) \approx \frac{n_{c}}{\sqrt{n_{1}}\sqrt{n_{2}}} \times Cor(Y_{1},Y_{2}),   $$

where in practice we need to estimate *Cor*(*Y*_1_,*Y*_2_) externally. A plot of Eq. 7 is given in Additional file 1: Figure S1.

### Correction for overlapping samples in case-control studies

When the data refer to two case-controls studies we give the result previously derived by Lin and Sullivan [12]. Let *n*_*c*0_ denote the number of overlap in controls in study 1 and 2, and *n*_*c*1_ denote the number of overlap for cases. First we derive *Cov*(*U*_1_(*β*_1_),*U*_2_(*β*_2_)) using the score function from logistic regression, and the fact that *y*_*ki*_=0 for cases and *y*_*ki*_=1 for controls

8 $$ {\selectfont{\begin{aligned} {}Cov (U_{1}(\beta_1), U_{2}(\beta_2)) & = \frac{1}{n_{C}} \sum\limits_{i \in S_{C}} x_{1i} x_{2i} \\ &\quad \times \left\{ \sum\limits_{i \in S_{C0}} \!\left(0 \,-\, \frac{\exp\{\alpha_{1} \}}{1\,+\,\exp\{\alpha_{1} \}}\!\right) \!\left(\!0 \,-\, \frac{\exp\{\alpha_{2} \}}{1+\exp\{\alpha_{2} \}}\right)\right. \\ & \quad \left.+ \sum\limits_{i \in S_{C1}} \left(\!1\! \,-\, \!\frac{\exp\{\alpha_{1} \}}{1+\exp\{\alpha_{1} \}}\right) \!\left(\!1 \,-\, \frac{\exp\{\alpha_{2} \}}{1\,+\,\exp\{\alpha_{2} \}}\!\right) \!\right\}. \end{aligned}}}  $$

It is easy to show that the right hand side of 8 is equal to $\frac {1}{(1+\exp \{\alpha _{1}\})(1+\exp \{\alpha _{2}\})} \big \{ n_{c0}\exp \{(\alpha _{1}+\alpha _{2})\}+n_{c1} \big \} \frac {1}{n_{c}} \sum \limits _{i \in S_{C}} x_{1i} x_{2i}$. According to assumption 2 we can introduce *H* to obtain $Cov (U_{1}(\beta _{1}), U_{2}(\beta _{2})) = \frac {1}{(1+\exp \{\alpha _{1}\})(1+\exp \{\alpha _{2}\})} \big \{ n_{c0}\exp \{(\alpha _{1}+\alpha _{2})\}+n_{c1} \big \} H$. In logistic regression under the null model there is a connection between the intercept and the log odds $\exp \{\alpha _{k}\} = \frac {n_{kc0}}{n_{k}} / \left (1-\frac {n_{kc0}}{n_{k}}\right) =n_{kc0}/n_{kc1}$.

From Eq. 5, it follows that

9 $$ {\selectfont{\begin{aligned} {}\text{Cor}\left(\hat{\beta}_{1}, \hat{\beta}_{2}\right) \!\approx\! \frac{1}{\sqrt{n_{1}}\sqrt{n_{2}}} \!\times\! \left(n_{c0}\sqrt{\exp \{\alpha_{1} + \alpha_{2}\}} + \frac{n_{c1}}{\sqrt{\exp \{\alpha_{1} + \alpha_{2}\}}} \right). \end{aligned}}}  $$

### Correction for overlapping samples with one quantitative trait study and case control study

Finally, we consider one *Y*_1_ quantitative and *Y*_2_ binary. In Eq. (5) we use the score function and the Fisher information derived in both the logistics and linear regression model and arrive at

10 $$\begin{array}{@{}rcl@{}} Cor \left(\hat{\beta}_{1}, \hat{\beta}_{2}\right) &\approx& \left(\frac{1}{\sigma^{2}_{1}} \sum\limits_{i \in S_{1}} x_{1i} x_{1i}\right)^{-1/2}  \\ & &\times \frac{1}{\sigma^{2}_{1}} \frac{1}{n_{12}} \sum\limits_{i \in S_{C}} (y_{1i} -(\alpha_{1}))(y_{2i} -p_{2}) x_{1i} x_{2i}  \\ & & \times \left(p_{2}(1-p_{2})\sum\limits_{i \in S_{2}} x_{2i} x_{2i}\right)^{-1/2}, \end{array} $$

where *p*_2_ is the proportion of cases in the case control study. Substituting in *H*, $Cor \left (\hat {\beta }_{1}, \hat {\beta }_{2}\right) \approx \left (\frac {1}{\sigma ^{2}_{1}} n_{1} H\right)^{-1/2} \times \frac {1}{\sigma _{1}^{2}}H \sum \limits _{i \in S_{C}} (y_{1i} - \alpha _{1})(y_{2i}-p_{2}) \times (p_{2}(1-p_{2})n_{2} H)^{-1/2} $. This can be approximated as $Cor\left (\hat {\beta }_{1}, \hat {\beta }_{2}\right) \approx \frac {n_{c}}{\sqrt {n_{1} \cdot n_{2}}} \text {Cor}_{pb}(Y_{1}, Y_{2}) $, where Cor_*pb*_(*Y*_1_,*Y*_2_) is the point-biserial correlation coefficient which needs to be estimated externally when only summary statistics are available.

### Decorrelation

The focus here is correcting the bivariate distribution of GWAS test statistics for the correlation due to sample overlap. The test statistics may come from case-control studies or studies on quantitative traits. We also assume that the effect direction is known and that the summary statistics are given as Wald statistics, i.e. $\hat {\beta }_{k}/se\left (\hat {\beta }_{k}\right)$, where $se\left (\hat {\beta }_{k}\right)$ is the standard error for the regression coefficient of every SNP g, where as before we drop g from the notation. For large samples, Wald statistics approximately follow a standard normal distribution and as such are interpretable as *z*-scores.

Thus, our final data-set is a matrix **z** consisting of two rows and *d* columns equal to the number of SNPs common to both studies, including the vector of *z*-scores for the first study, *z*_1_, in the first row and the vector of *z*-scores for the second study, *z*_2_, in the second row.

To correct for the overlap in samples and to remove the spurious correlation from the data we use a decorrelation transformation as described by [26]. The transform is defined as

11 $$  \mathbf{z_{\text{de-corr}}} = \mathbf{C}^{-1/2} \mathbf{z}  $$

where **C** is the 2×2 empirical correlation matrix of **z**, with *r*=*cor*(*z*_1_,*z*_2_) on its off-diagonal. Note this is different from the Mahalanobis transform, which uses the covariance matrix in Eq. 11 instead of the correlation matrix **C**. After the transformation, the correlation matrix of *z*_de-corr_ is a diagonal matrix. Importantly this transformation maximizes the correlation between the original data and the transformed data and is thus the most suitable transformation as it has the least impact on the data when performing pre-whitening [26].

Suppose that we want to decorrelate the test statistics of quantitative trait studies 1 and 2 but only for the amount of correlation due to sample sharing. Under the null hypothesis that a certain SNP *g* has no effect on the outcome in both studies, we know that $\text {cor}\left (\hat {\beta }_{1}, \hat {\beta }_{2}\right)$ is given by Eq. 1 and this correlation is purely induced by sample sharing. We want to correct exactly for this spurious correlation. It can be shown that for sufficiently large *n*_1_ and $n_{2} \text {cor}\left (\hat {\beta }_{1}, \hat {\beta }_{2}\right) \approx cor(z_{1}, z_{2})$. Then under the null hypothesis we should correct **z** with

12 $$  \mathbf{C_{adj}} = \left(\begin{array}{cc} 1 &\frac{n_{c}}{\sqrt{n_{1} \cdot n_{2}}} cor(Y_{1}, Y_{2}) \\ \frac{n_{c}}{\sqrt{n_{1} \cdot n_{2}}} cor(Y_{1}, Y_{2}) & 1 \end{array} \right)  $$

assuming the **y**_*k*_ are quantitative traits. Alternatively, **C** could be calculated using the methods of [25] or [14] if lacking explicit information on the number of overlapping subjects.

### Simulation study

**Simulation of genotype and phenotype** For all scenarios, we simulated *d*=100,000 independent SNPs with a MAF drawn at random from the observed distribution of MAF from the 1000 Genomes Project. The quantitative trait outcomes, *Y*_1_ (study 1 outcome) and *Y*_2_ (study 2 outcome), were simulated for *n*=20,000 individuals, *n*_1_=*n*_2_=10,000 individuals per study.

The six simulation scenarios differ in the simulation of the outcomes. For the null model, we simulate *Y*_1_ and *Y*_2_ as described in the example in the “Methods” section.

For all other simulation scenarios, *Y*_1_ and *Y*_2_ are dependent on both the error term and a given subset of SNPs. For the “positive pleiotropy A” scenario, the signal involves SNPs that are non-null for both *Y*_1_ and *Y*_2_. We set 400 regression parameters not equal to zero (*β*=0.1 for 100 SNPs, *β*=−0.1 for 100 SNPs, *β*=0.15 for 100 SNPs, and *β*=−0.15 for 100 SNPs) with the same effect strength and direction on *Y*_1_ and *Y*_2_. This gives 400 non-null SNPs and 99,600 null SNPs for both study 1 and study 2. Similarly for the “positive pleiotropy B” scenario, we increase the polygenicity and set 1200 regression parameters not equal to zero (*β*=0.1 for 100 SNPs, *β*=−0.1 for 100 SNPs, *β*=0.07 for 500 SNPs, and *β*=−0.07 for 500 SNPs) with the same effect strength and direction on *Y*_1_ and *Y*_2_. For the “positive pleiotropy C” scenario, we increase the polygenicity again and set 2200 regression parameters not equal to zero (*β*=0.1 for 100 SNPs, *β*=−0.1 for 100 SNPs, *β*=0.05 for 1000 SNPs, and *β*=−0.05 for 1000 SNPs) with the same effect strength and direction on *Y*_1_ and *Y*_2_.

For the “positive pleiotropy plus univariate effects in study 1” scenario, we introduce positive pleiotropy by setting 200 regression parameters not equal to zero (*β*=0.1 for 100 SNPs, *β*=−0.1 for 100 SNPs) with the same effect strength and direction on *Y*_1_ and *Y*_2_. Additionally, we add a signal for 200 SNPs that is only present in study 1 (*β*=0.15 for 100 SNPs, *β*=−0.15 for 100 SNPs). In the final simulation scenario, we generate “positive and antagonistic pleiotropy” by setting 200 regression parameters not equal to zero (*β*=0.1 for 100 SNPs, *β*=−0.1 for 100 SNPs) with the same effect strength and direction on *Y*_1_ and *Y*_2_, and additionally, we add 200 SNPs with opposing effect directions for study 1 and study 2 (*β*_1_=0.15 and *β*_2_=0.15 for 100 SNPs, *β*_1_=−0.15 and *β*_2_=0.15 for 100 SNPs).

**Generation of independent and overlapping studies** For each simulation scenario, we computed GWAS summary statistics for the ideal case of two studies with no overlap in samples. We refer to these as *independent studies*. Additionally, for each simulation scenario, we generated summary statistics for studies with *n*_*c*_=5000 overlapping samples. In practice, we did this by randomly assigning 2500 subjects from study 1 to be included into study 2, and vice versa, resulting in *n*_1_=*n*_2_=12,500. These studies are referred to as the *overlapping studies*. Since the overlapping studies have more power than the independent studies, we also simulated independent studies with *n*_1_=*n*_2_=12,500 and refer to this as the *independent studies with equal power*.

In order to look at the effect of various amounts of sample overlap, we did an extended simulation using the “positive pleiotropy A” scenario, where the number of overlapping samples ranged from 500 to 5000, in steps of 500. In practice, we did this by randomly assigning 250,500,750,1000,…,2500 subjects from study 1 to be included into study 2, and vice versa. Thus the total overlap in samples adds up to *n*_*c*_=500,1000,1500,2000,…,5000 subjects, and the sample size per group is *n*_1_=*n*_2_=10250,10500, 10750,1100,…,12500.

In practice the correlation due to overlap may be subject to some estimation error. In order test the robustness of the proposed correction, we varied the correlation value used in the de-correlation step for the "positive pleiotropy A” scenario. For this simulation, the correlation due to overlap is 0.4 but we varied the correlation value in the de-correlation step from 0.3 to 0.5.

**Generation of GWAS test statistics and covariate modulated fdr** For each simulation scenario, separately for each of study 1 and 2 (“independent”) and again for each of study 1 and 2 (“overlapping”), we computed for each of the *d*=100,000 SNP we computed a univariate linear regression and estimate the effect size of each SNP by the *z*-score defined as regression coeffiecient divided by its standard deviation. These *z*-scores are the final summary statistics used in further analysis. The summary statistics were then used to calculate the cmfdr for study 1 using the study 2 summary statistics as the covariate. This was done first for the independent studies and then again using the overlapping studies. The summary statistics for the overlapping studies were then corrected using Eqs. 11 and 12 (“corrected”). The number of true positives (TP), false positives (FP) and the false discovery proportion (FDP) were calculated using a cmfdr cutoff of 0.05.

For each of the simulation scenarios described above, we performed 100 replicates and report the average TP, FP and FDP for the following three settings

1. independent study 1 and 2
2. uncorrected overlapping study 1 and 2
3. overlapping study 1 and 2 with the proposed correction

We define true positives as those SNPs where we introduced effects into the simulation, i.e. known non-null SNPs.

### Psychiatric genetics consortium application

**Data description** We were granted access to the raw genotype data for bipolar disorder cases, schizophrenia cases and controls from the Psychiatric Genetics Consortium (PGC) [21, 22]. The relevant institutional review boards or ethics committees approved the research protocol of the individual GWAS included in the PGC sample and all participants provided written informed consent. We used the PGC data to test the performance of our proposed correction for sample overlap in a real data setting, where we varied the amount of overlap in the control group between the schizophrenia and bipolar studies.

The data consists of *n*=9379 schizophrenia cases, *n*=6990 bipolar disorder cases and *n*=21,153 shared controls. Imputed genotypes in dosage format were available genome-wide, but we limited our analysis to 260,703 SNPs with *MAF*≥0.05 on chromosomes 1, 2 and 3 due to computational time. Using this dataset, we randomly selected 10,000 controls for schizophrenia, and then randomly selected 10,000 controls for bipolar disorder, of which 0, 2000, 4000, 6000, 8000 or 10000 were drawn from the schizophrenia controls, corresponding to an expected correlation of *ρ*=0,0.09,0.18,0.27,0.36,0.45 respectively between the GWAS summary statistics for bipolar disorder and schizophrenia. We repeated each of these conditions 10 times. We then conducted a standard GWAS for each of the 120 datasets (6 amounts of overlap * 2 types of cases * 10 repetitions) by conducting logistic regression in Plink (v1.07), adjusting for population stratification using the first two principle components. We then took the summary statistics from each GWAS and entered them pairwise into the cmfdr using the bipolar disorder summary statistics as the covariate for schizophrenia. The cmfdr calculations were done for both the raw data and also for the data after correction for sample overlap.

## Additional file

Additional file 1 Plot of correlation due to overlap versus quantitative trait correlation. Supplemental Figure 1. Plot of the correlation due to overlap for two quantative traits as a function of percent sample overlap and the correlation of the traits (*Cor*(*Y*_1_,*Y*_2_)). Here we assume the sample sizes for the two GWASs are equal. The See Eq. 7. (PDF 40 kb)
