# Supplementary figures and images for: A correction for sample overlap in genome-wide association studies in a polygenic pleiotropy-informed framework
Source: BMC Genomics. 2018 Jun 25;19:494. doi: 10.1186/s12864-018-4859-7 (PMC6019513; doi:10.1186/s12864-018-4859-7)

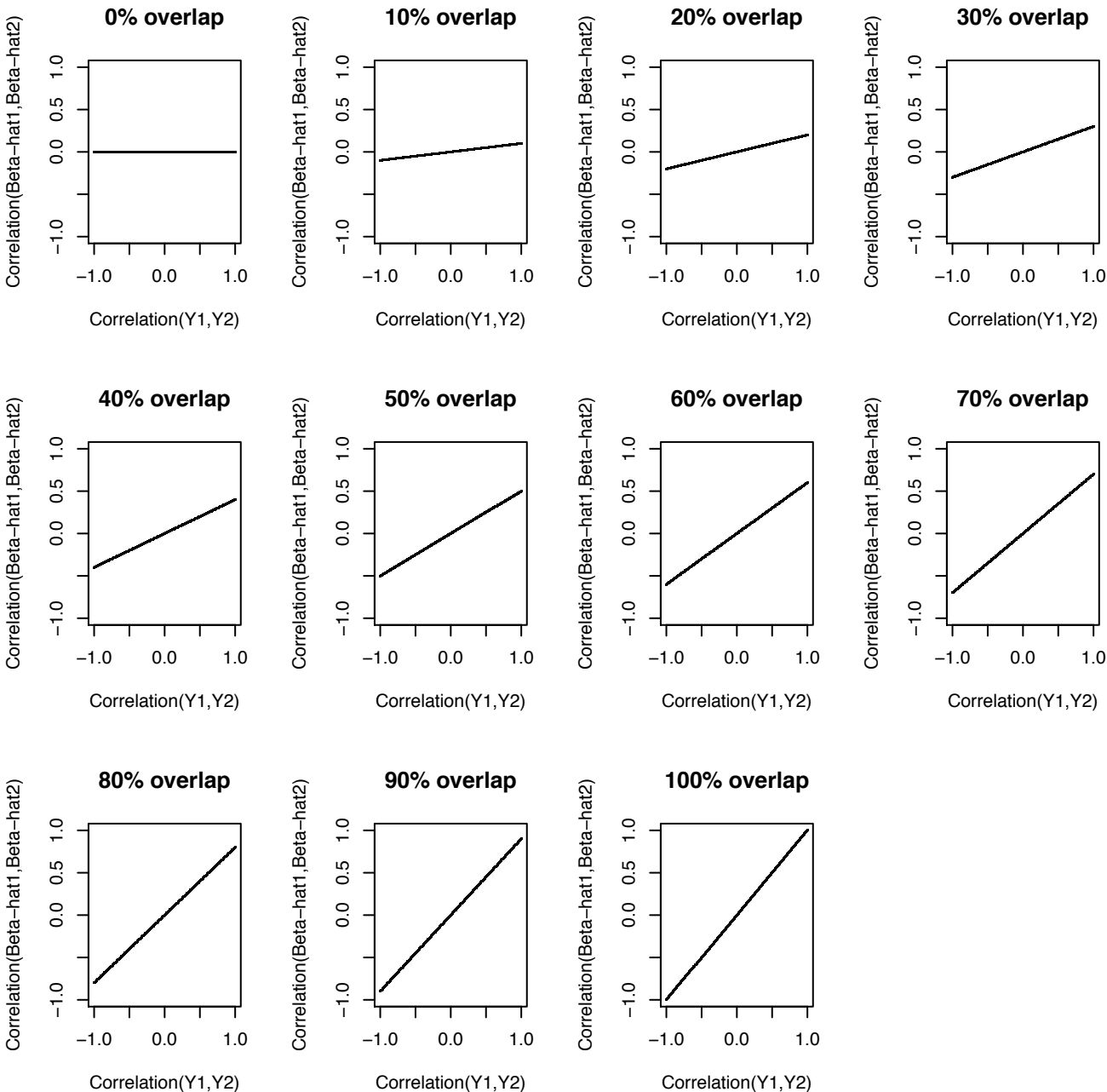

Supplement: Supplementary file 1 — Plot of correlation due to overlap versus quantitative trait correlation. Supplemental Figure 1. Plot of the correlation due to overlap for two quantative traits as a function of percent sample overlap and the correlation of the traits (Cor(Y1,Y2)). Here we assume the sample sizes for the two GWASs are equal. The See Eq. 7. (PDF 40 kb) [file 12864_2018_4859_MOESM1_ESM.pdf]
